# The Novel Triazolonaphthalimide Derivative LSS-11 Synergizes the Anti-Proliferative Effect of Paclitaxel via STAT3-Dependent MDR1 and MRP1 Downregulation in Chemoresistant Lung Cancer Cells

**DOI:** 10.3390/molecules22111822

**Published:** 2017-10-26

**Authors:** Liyan Ji, Xi Liu, Shuwei Zhang, Shunan Tang, Simin Yang, Shasha Li, Xiaoxiao Qi, Siwang Yu, Linlin Lu, Xiangbao Meng, Zhongqiu Liu

**Affiliations:** 1 International Institute for Translational Chinese Medicine, Guangzhou University of Chinese Medicine, Guangzhou 510006, China; liyanji@gzucm.edu.cn (L.J.); liuxi20150710@163.com (X.L); zswatobe@163.com (S.Z.); qixiaoxiao@gzucm.edu.cn (X.Q.); lllu@gzucm.edu.cn (L.L.); 2 The Postdoctoral Research Station, Guangzhou University of Chinese Medicine, Guangzhou 510006, China; 3 Department of Chemical Biology, School of Pharmaceutical Sciences, Peking University, Beijing 100191, China; tangshunan1990@126.com (S.T.); yangsimin16@163.com (Sim.Y.); shashali@umich.edu (S.L.); swang_yu@bjmu.edu.cn (Siw.Y.)

**Keywords:** triazolonaphthalimide derivative, paclitaxel resistance, lung cancer, STAT3 inhibition

## Abstract

Multidrug resistance (MDR) is a major cause of the inefficacy and poor response to paclitaxel-based chemotherapy. The combination of conventional cytotoxic drugs has been a plausible strategy for overcoming paclitaxel resistance. Herein, we investigated the cytotoxic effects and underlying mechanism of **LSS-11**, a novel naphthalimide derivative-based topoisomerase inhibitor, in paclitaxel-resistant A549 (A549/T) lung cancer cells. **LSS-11** enhanced cell death in A549/T cells by inducing apoptosis through increasing the DR5 protein level and PARP1 cleavage. Importantly, **LSS-11** dose-dependently reduced STAT3 phosphorylation and downregulated its target genes MDR1 and MRP1, without affecting P-gp transport function. Chromatin coimmunoprecipitation (ChIP) assay further revealed that **LSS-11** hindered the binding of STAT3 to the MDR1 and MRP1 promoters. Additionally, pharmacological inhibition of p-STAT3 by sulforaphane downregulated MDR1 and MRP1, resulting in A549/T cell death by triggering apoptosis. Collectively, our data show that **LSS-11** is a potent naphthalimide-based chemosensitizer that could enhance cell death in paclitaxel-resistant lung cancer cells through the DR5/PARP1 pathway and STAT3/MDR1/MRP1 STAT3 inhibition.

## 1. Introduction

Lung cancer is the highest mortality of all types of cancer worldwide, causing 27% of cancer deaths [1]. Taxane-based chemotherapies are used to treat approximately 70% of lung cancer patients [2,3]. Unfortunately, 40% of these patients recur or relapse after initial treatment as a result of acquired resistance [4]. Reversal of paclitaxel resistance is thus a major concern for clinical and experimental oncologists [5,6].

Elevated multiple resistance-associated transporters have been considered the major contributors to paclitaxel resistance in lung cancer [7]. Acquired de novo and paclitaxel resistance could be mainly ascribed to transporters encoding by ATP-binding cassette (ABC) genes [8]. The ABC transporter family contain 49 members divided into seven subfamilies in humans. At least thirteen ABC genes are involved in drug resistance, including MDR1/ABCB1, BCRP/ABCG2, MRPs1-9/ABCCs1-9 [9]. Among them, MDR1/ABCB1, also known as p-glycoprotein (P-gp), are transporters or inhibitors of 324 approved drugs and investigational candidates in the DrugBank database [10], and they facilitate the transmembrane transport of paclitaxel and other anti-tumor drugs as well as rhodamine 123 DNA dyes. The MRPs (1–3) also drive resistance to paclitaxel and other natural anti-tumor drugs [9]. The consecutive expression or inducible expression of MRPs is controlled by transcription factors (e.g., Sp1, p53, HIF-1, and Nrf2), nuclear receptors (e.g., PXR, CAR, and RXR) and kinases (e.g., PI3K/Akt) [11]. Among them, STAT3 is a critical transcription factor for transcriptional regulation of MDR1 and MRP1 gene expression [12,13]. Inactivation of STAT3 overcomes taxane resistance in various cancers, including lung cancer cells [14]. Thus, suppression of STAT3 phosphorylation might sensitize MDR-overexpressing lung cancer cells to paclitaxel. 

Recently, non-transporters termed monoresistance genes such as topoisomerase, HDAC1, and β-III-tubulin have been found to confer intrinsic resistance to paclitaxel in lung cancer cells [15]. Topoisomerase IIα functions by decatenating double-stranded DNA and disentangling chromatin during DNA replication and transcription, and serves as a proliferative marker in tumor cells [16]. Topoisomerase IIα overexpression correlates with poor survival in chemotherapy treated non-small cell lung cancer patients [17]. Topoisomerase inhibitors have been combined with paclitaxel in lung cancer treatment [18]. However, most topoisomerase inhibitors (e.g., doxorubicin and amrubicin) are substrates for P-gp, resulting in multiple drug cross-resistance and consequently treatment failure [19,20]. Therefore, searching for novel chemosensitizers is a feasible and effective approach to overcome paclitaxel-resistance in lung cancer.

Naphthalimide derivatives show highly active toxicity against a variety of tumors, both in vitro and in vivo, as classic topoisomerase inhibitors [21]. Intriguingly, some of these chemicals also have been reported to overcome drug resistance in various neoplasms, including hematological cancer [22] and solid tumors [23]. One naphthalimide was reported to be neither a substrate nor an inhibitor of p-glycoprotein, MRP1, or BCRP [24]. Another one, **DMP-840**, exhibited no cross-resistance to vincristine in xenograft mice and to topotecan in rhabdomyosarcomas [23]. However, whether naphthalimide derivatives could sensitize resistant-lung cancer cells to paclitaxel remains unclear.

**Figure 1:**
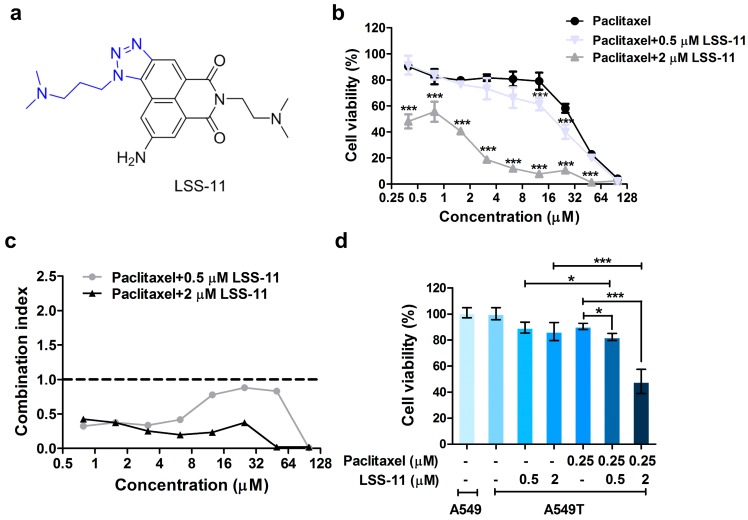
**LSS-11** overcomes paclitaxel-resistant in A549/T cells. (**a**) Chemical structure of LSS-11; (**b**) Cell proliferation inhibition of A549/T cells in the presence of paclitaxel plus **LSS-11** measured by MTT method for 72 h. Data are shown as mean ± s.d. (*n* = 6), *** *p* < 0.001 by Student’s *t* test; (**c**) Combination index of **LSS-11** and paclitaxel in A549/T; (**d**) Cell viability of A549 or A549/T cells treated with or without drugs. Data are shown as mean ± s.d. (*n* = 6), * *p* < 0.05, *** *p* < 0.001.

We previously synthesized a series of triazolonaphthalimide moieties with selective anti-tumor activity ranging from the nanomolar to the micromolar range [25]. In the present study, we investigated the effect of **LSS-11** (9-amino-6-(2-dimethylamino)propyl]-1-(3-(dimethylamino)-propyl)benzo[de][1,2,3]triazolo[5,4-*g*]isoquinoline-5,7(1*H*,6*H*)-dione; Figure 1a; for its synthesis, see Supplementary Scheme 1), a potent topoisomerase inhibitor with DNA minor groove-binding activity [26], on paclitaxel-resistant lung cancer cells and its underlying mechanism. We found that **LSS-11** enhanced apoptosis in paclitaxel-resistant lung cancer cells, indicating that triazolonaphthalimide could be a potential agent for overcoming paclitaxel-resistance in lung cancer. Importantly, inactivation of STAT3 by **LSS-11** repressed the gene expression of MRPs, triggering apoptosis to make resistant lung cancer cells more susceptible to chemotherapy. Our findings provide a novel compound for potential use in combination regimens following paclitaxel in lung cancer treatment.

## 2. Results

### 2.1. ***LSS-11*** Reverses Paclitaxel Resistance in Lung Cancer Cells

To determine whether **LSS-11** could overcome paclitaxel resistance, we measured cell proliferation to evaluate the effect of paclitaxel in the conjugation of **LSS-11** in paclitaxel-resistant A549 (A549/T) cells. MTT assay on cells treated with 0.5 and 2 μM **LSS-11** alone showed A549/T cell viabilities of 86% and 85%, respectively, for 72 h. As shown in Table 1, the IC_50_ of paclitaxel alone was 36.32 ± 8.29 µM, with a resistance index of 19 in A549/T cells, compared with 1.9 ± 0.36 µM in A549 cells. The IC_50_ of **LSS-11** was 6.87 ± 0.77 μM and > 10 μM in A549/T and A549 cells, respectively (Table 1 and Supplementary Figure S1). When combined with **LSS-11** (2 µM), paclitaxel induced a significantly lower cell viability than paclitaxel alone in A549/T cells (all *p* < 0.001; Figure 1b). Furthermore, all combination-index values were lower than 1 (synergism) in the combination of paclitaxel (0~100 µM) with **LSS-11** (0.5 and 2 µM) (Figure 1c), suggesting that LSS-11 could overcome the paclitaxel resistance in A549/T cells.

To exclude synergistic effects by target conjugation, we used concentrations (0.5 and 2 µM) of **LSS-11** with survival ratios >85% in subsequent experiments (Figure 1d). The cell viabilities varied from 90.46 ± 2.39% in A549/T cells treated with paclitaxel alone to 82.28 ± 2.70% and 48.20 ± 9.30%, respectively, in the conjugation of paclitaxel with **LSS-11** at 0.5 and 2 µM (Figure 1d). Consistent with the combination index, cells treated with **LSS-11** combined with paclitaxel exhibited remarkably lower cell viabilities than A549 cells (all *p* < 0.001) and those exposure to **LSS-11** alone (*p* < 0.05 for 0.5 µM and *p* < 0.001 for 2 µM). These data indicate that **LSS-11** sensitized paclitaxel-resistant lung cancer cells to paclitaxel.

### 2.2. ***LSS-11*** Augments the Pro-Apoptotic Effect in Paclitaxel-Resistant Lung Cancer Cells 

**Figure 2:**
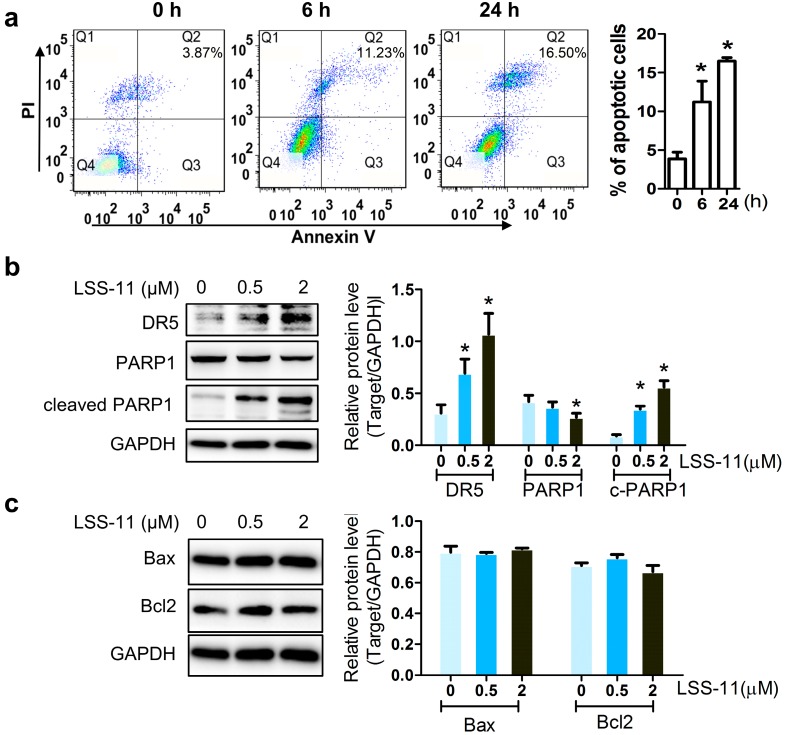
**LSS-11** enhances cell apoptosis in A549/T cells. (**a**) Percentage of apoptotic A549/T cells were analyzed by flow cytometry after exposure to 0.5 μM LSS-11 at indicated times. Protein levels of DR5, PARP-1, cleaved PARP1 (**b**), Bax, and Bcl2 (**c**) after **LSS-11** treatment for 24 h. Quantification of protein bands was shown as bar graph in the right panels. * *p* < 0.05 using Student’s *t* test.

To investigate the pathway involved in cell proliferation inhibition enhanced by **LSS-11**, we measured apoptotic markers in A549/T cells. Annexin V/PI staining was used to assess the percentage of apoptotic cells induced by paclitaxel in combination with **LSS-11**. As shown in Figure 2a, the percentage of apoptotic cells represented by Annexin V^+^/PI^+^ cells (Q2) quarter was significantly elevated from 3.87 ± 1.48% to 11.23 ± 3.78% and 16.50 ± 0.79% after 0.5 µM LSS-11 treatment for 6 h and 24 h in A549/T cells. Moreover, **LSS-11** dose-dependently increased DR5 and cleaved PARP1, while **LSS-11** had no significant effect on the protein levels of Bax or Bcl2 in A549/T cells, compared with vehicle (0.1% DMSO) treated cells (Figure 2b,c). These observations indicate that **LSS-11** enhanced paclitaxel-resistant cell death via DR5/PARP1-mediated apoptosis.

### 2.3. ***LSS-11*** Has No Effect on P-gp Efflux Function in Drug-Insensitive A549/T Cells

Increased drug efflux by transporters such as P-gp and MRP1 results in reduced drug concentration and ultimately resistance to paclitaxel in lung cancer [27]. To investigate whether the enhancement of paclitaxel-induced cell death by **LSS-11** is mediated by inhibiting the function of P-gp, rhodamine 123 efflux was detected by flow cytometry. The results showed that the fluorescence intensity of rhodamine 123 was not significant in both **LSS-11**-treated A549 and A549/T cells compared with control cells (Supplementary Figure S2a,b). This indicates that **LSS-11** is not a substrate of P-gp.

### 2.4. ***LSS-11*** Suppresses mRNA Levels of Drug Resistance Genes in A549/T Cells

**Figure 3:**
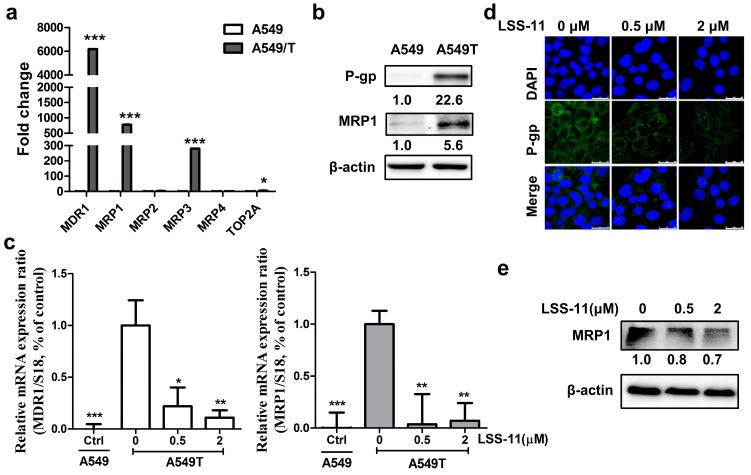
**LSS-11** downregulates multiple drug resistance genes in A549/T cells. (**a**) mRNA levels of drug resistance genes in A549 and A549/T cells were detected by qPCR; (**b**) Western blot analysis of P-gp and MRP1 in A549 and A549/T cells; (**c**) Gene expression of MDR1 and MRP1 induced by **LSS-11** for 24 h. * *p* < 0.05; ** *p* < 0.01; *** *p* < 0.001, compared with vehicle treated A549/T cells; qPCR results were performed in technical triplicates for each sample; (**d**) Representative image of immunofluorescence demonstrated that **LSS-11** reduced P-gp expression in A549/T cells. Nuclei and P-gp were stained with DAPI (blue) and FITC-linked antibody (green); (**e**) Immunoblotting was used to test the protein level of MRP1 in A549/T cells treated with **LSS-11** for 24 h.

Increased MRPs levels also contribute to chemoresistance [28]. To explore the transcriptional levels of drug resistance genes in paclitaxel resistance, we first detected the baseline mRNA levels of MDR1, MRP1-4, and TOP2A in A549/T and A549 cells qPCR (Figure 3a). The gene expression of MDR1, MRP1, and MRP3 increased 6170-fold, 780-fold, and 280-fold, respectively, in A549/T cells compared with A549 cells, while MRP2 and MRP4 exhibited no significant change between A549 and A549/T cells (Figure 3a and Supplementary Figure S3). We also found that TOP2A increased six-fold in A549/T cells compared with A549 cells (Figure 3a). Analogously to the gene expression data, increases of expression in the two predominant proteins, P-gp (coding by MDR1) and MRP1, were observed in A549/T cells (Figure 3b), whereas the protein level of MRP3 was inconsistent with its gene expression (data not shown). These data indicate that MDR1 and MRP1 are two predominant drug-resistant genes between A549/T cells and A549 cells and that they might contribute to paclitaxel resistance in lung cancer cells. 

Next, to illustrate how **LSS-11** affected the gene expression of predominant drug resistance genes, we measured the gene expression levels of MDR1 and MRP1 in **LSS-11** treated A549/T cells. The result revealed that 0.5 μM **LSS-11** dramatically downregulated the expression of MDR1 and MRP1 by 9-fold and 26-fold, respectively, in A549/T cells (Figure 3c), whereas MRP3 was not significantly altered by **LSS-11** in A549/T cells (data not shown). Furthermore, the protein abundance of P-gp was lower in A549/T cells treated with **LSS-11** than vehicle cells as measured by immunofluorescence (Figure 3d). In addition, protein expression of MRP1 significantly decreased in A549/T cells treated with 2 μM **LSS-11** (Figure 3e).

### 2.5. ***LSS-11*** Downregulates MDR1 and MRP1 via Repression of STAT3 in A549/T Cells

**Figure 4:**
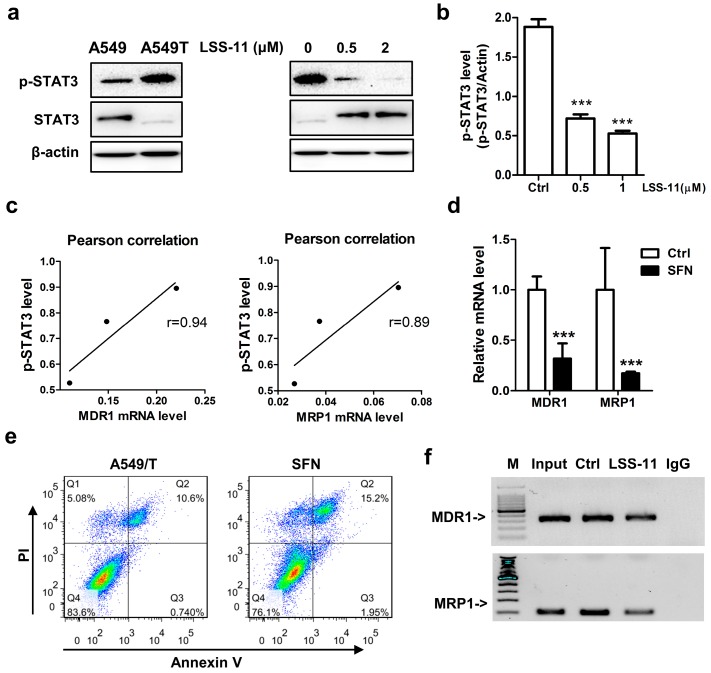
**LSS-11** inhibits STAT3 phosphorylation and hinders STAT3 binding to MDR1 and MRP1 promoters. (**a**) Western blot analysis of p-STAT3 and STAT3 in A549 and A549/T cells treated with or without **LSS-11** for 24 h. β-actin was used as loading control; (**b**) Quantification of immunoblot bands of p-STAT3 and STAT3 by ImageJ software. *** *p* < 0.001; (**c**) Pearson correlation efficient between suppression of p-STAT3 and downregulation of MDR1 or MRP1 by **LSS-11;** (**d**) Gene expression of MDR1 and MRP1 induced by STAT3 inhibitor sulforaphane. *** *p* < 0.001; (**e**) Apoptotic cells in A549/T cells treated with STAT3 inhibitor sulforaphane (20 µM) for 24 h as measured by annexin V/PI staining; (**f**) STAT3 ChIP-qPCR analysis displayed the binding sites of STAT3 in the promoter region of MDR1 (upper) and MRP1 (lower) genes. All experiments were performed in triplicates.

Since the gene expression of drug resistance genes is controlled by regulators, it is reasonable that transcription factors lie within the promoter region of both MDR1 and MRP1 could be affected by **LSS-11**. We searched through ChIP data in the ENCODE database and found that transcription factors (TFs) including STAT3, Nrf2, PXR, and ERα putatively bound to both MDR1 and MRP1 promoter regions. Thus, we conducted western blots to probe the upstream TFs that **LSS-11** targeted to downregulate MDR1 and MRP1 in A549/T cells. Interestingly, **LSS-11** significantly reduced the phosphorylated level of STAT3, while it increased the total protein level of STAT3 (Figure 4a,b; *p* < 0.001), but it showed no significant influence or consistent changes on other tested regulators at either total or nuclear protein levels (Supplementary Figure S4). Additionally, the suppression of phosphorylated-STAT3 was well-correlated with the fold change of MDR1 and MRP1 gene (*r* = 0.94 and *r* = 0.89, respectively; Figure 4c). Sulforaphane (SFN) [29], a pharmacological inhibitor of STAT3 signaling, downregulated the mRNA levels of MRP1 and MDR1, similar to **LSS-11** (Figure 4d). As expected, the percentage of apoptotic cells were more pronounced in sulforaphane (20 µM) treated cells compared to paclitaxel-resistant A549/T cells as measured by Annexin V/PI staining (average 14.45 ± 1.06% for SFN vs 9.20 ± 1.05% for control; Figure 4e).

To further prove whether the downregulation of MDR1 and MRP1 by **LSS-11** was directly mediated via STAT3, ChIP-qPCR was performed in A549/T cells. The results showed that **LSS-11** attenuated the binding of STAT3 to both the MDR1 and MRP1 promoter regions (Figure 4f). Collectively, these data indicated that downregulation of MRP1 and MDR1 by **LSS-11** is mediated by STAT3 inactivation.

## 3. Discussion

In our present study, a newly synthesized triazolonaphthalimide, **LSS-11**, was assessed for its effect on the sensitivity of A549/T cells to paclitaxel. A549/T cells showed resistance to paclitaxel, as validated by their resistance index of 19. In contrast, A549 and A549/T cells had almost an identical IC_50_ values, suggesting that A549/T cells were still sensitive to **LSS-11** treatment. **LSS-11** in conjugation with paclitaxel enhanced the cytotoxic effect of paclitaxel in A549/T cells, suggesting **LSS-11** could overcome the resistance to paclitaxel in lung cancer cells (Figure 1c). We also showed that their synergistic effect might be due to downregulation of multiple drug resistance genes through STAT3 inhibition.

We found that MDR1 and MRP1 were the predominant paclitaxel-resistance genes in A549/T cells, and **LSS-11** dramatically downregulated MDR1 and MRP1 in A549/T cells without affecting P-gp function. Consistent with a previous report, we observed that MDR1, MRP1, and TOP2A were frequently upregulated in paclitaxel resistance [30]. Unlike the previous study [31], MRP2 and MRP4 were not associated with acquired resistance to paclitaxel in NSCLC cells. Notably, MDR1 and MRP1 with ten-to-thousands-fold upregulation, were the main paclitaxel resistance genes in lung cancer cells (Figure 3a). Notably, the naphthalimide-derivative **LSS-11** downregulated MRP1 and MDR1 in A549/T cells (Figure 3b,c). Unlike most MDR inhibitors, the synergistic effect of **LSS-11** with paclitaxel was partially attributed to downregulation of multiple drug resistance genes rather than P-gp inhibition.

Chemotherapy triggers kinase cascades (JAK/STAT3 and PI3K/Akt) that upregulate MRP1 to induce drug resistance [32]. In the present study, we found that chemotherapy initiated a STAT3 pathway to spur tumor cells resistant to drug stimuli. This notion is supported by the observation that paclitaxel activated STAT3 and upregulated MRP1 in lung cancer cells (Figure 3a and Figure 4a). STAT3 phosphorylation is necessary for its transcriptional activity and thus its role in chemotherapy resistance [33,34]. However, the functional role of Ser-727 STAT3 on transcriptional activity is controversial. Here, we showed that **LSS-11** inhibited STAT3 Ser-727 and potential transcriptional activity mimic SFN, a transcriptional inhibitor of STAT3 [35]. In agreement with a previous report [14], STAT3 inhibition by **LSS-11** partially leads to resistant lung cancer cells susceptible to paclitaxel. Conversely, STAT3 activation confers lung cancer resistant to paclitaxel [36]. This is partially caused by STAT3 targeted genes through binding consensus sequence in the promoter region of MDR1 to regulate its transcription [12]. Zhu et al. have reported MRP1 was downregulated after STAT3 inhibitor treatment [13], but the binding site of STAT3 to MRP1 was not clear. We searched for the transcription factor consensus sequences in the MRP1 promoter and found a predicted binding that would be triggered by exogeneous stimuli. We further provided direct evidence that STAT3 could bind to base pairs −504 to −398 upstream of the TSS of MRP1 to regulate its transcription in the presence of paclitaxel. Although there was a strong correlation (0.89~0.94) between downregulation of MDR1/MRP1 and inhibition of STAT3, other mechanisms might explain the downregulation of MDR1 and MRP1 by **LSS-11**. **LSS-11** treatment elevated total STAT3 level, but somehow decreased phosphorylation of STAT3. This kind of discrepancy indicate the complex effects of **LSS-11**. The comprehensive inhibitory effect of naphthalimide-derivatives on kinases including Akt [37], I-κB [38], and Chk2 [39], suggest that this class of compounds with a naphthalimide core could function as multi-kinase inhibitors. However, the direct inhibition of STAT3 phosphorylation and the effect on another phosphorylation site of STAT3-Tyr705 by **LSS-11** needs to be further addressed.

The STAT3-induced downregulation of MDR1 and MRP1 might increase the intercellular concentration of paclitaxel and subsequently trigger apoptosis in paclitaxel resistant lung cancer cells. Paclitaxel induces tumor cell death by G2/M phase arrest and apoptosis in sensitive cells [40]. DR5-dependent apoptosis augments cell death preferentially in resistant tumor cells [41] and recruits PARP1 in DR5-related death signaling [42]. In this study, **LSS-11** significantly increased the levels of DR5 and cleaved PARP1 (Figure 2b), proteins mainly involved in death-receptor-mediated apoptosis [43], but did not affect mitochondria-associated Bax or Bcl2 proteins in paclitaxel-resistant lung cancer cells. These findings indicate the DR5/PARP1 induced extrinsic apoptotic pathway could overcome paclitaxel resistance. However, whether the intrinsic apoptosis is induced by paclitaxel accumulation is currently under investigation.

## 4. Materials and Methods 

### 4.1. Reagents

**LSS-11** was synthesized by Meng’s laboratory (Peking University Health Center, Beijing, China). Paclitaxel was obtained from Dalian Meilun Co. (Dalian, China) and dissolved in DMSO to make a stock solution of 50 mM. Primary antibodies against DR5, PARP1, cleaved PARP1, Bax, Bcl2, GAPDH, and secondary antibodies including FITC-linked anti-rabbit and HRP-linked anti-rabbit or anti-mouse were obtained from Cell Signaling Technology (Danvers, MA, USA). Anti-P-gp (Santa Cruz, #sc-55510), anti-MRP1 (Santa Cruz, #sc-7774), anti-p-STAT3 (Ser 727, Santa Cruz, #sc-8001-R), and anti-STAT3 (Santa Cruz, #sc-482), were obtained from Santa Cruz Biotechnology (Santa Cruz, CA, USA). Chemiluminescent agents were purchased from Bio-Rad (Hercules, CA, USA). Annexin V/PI detection kit were obtained from BD Biosciences (San Diego, CA, USA). Ethidium bromide, propidium iodide (PI), rhodamine123 were brought from Sigma-Aldrich (St. Louis, MO, USA). 

### 4.2. Cell Culture

A549 cells were obtained from American Type Culture Collection (Manassas, VA, USA). A549/paclitaxel cells were donated by Liu’s laboratory MIAR (MUST, Macau, China) and cultured with medium containing constant concentration of paclitaxel (0.25 µM). Cells were cultured in RPMI 1640 medium containing 10% fetal bovine serum at 37 °C with 5% CO_2_ in a humidity circumstance.

### 4.3. MTT Assay

Cell proliferation was measured by MTT colorimetric method. Briefly, A549 and A549/T cells were seeded in 96-well plate in a density of 1 × 10^4^. Cells were treated with paclitaxel in the presence or absence of **LSS-11** for 24 h or 72 h. Then medium was discarded and 0.5 mg/mL MTT in 1 × PBS was added into each well. Cells were cultured for additional 2 h, then supernatant was removed and 150 μL DMSO was added into each well. Following the formazan mixed homogeneously, the absorbance was measured at 570 nm by microplate reader. 

### 4.4. Drug Combination

A549/T or A549 cells were seeded in 96-well plates in a density of 6000 cells per well. Following 24 h culture, cells were treated with **LSS-11** (0.001~10 μM) with and without paclitaxel (0.001~100 μM) for 24 h. Then, cell viability was measured by the MTT method. The drug combination index (CI) was calculated by the following function CI = (CA, x)/(ICx, A) + (cB, x)/(ICx, B) [41]. Where cA, x and cB, x were concentration of drug A, B when the combined inhibition rate was x%. While ICx, A, ICx, B represented the concentration of single drug A and B when inhibition rate of single agent was x%. The calculated value represents synergistic (CI < 1), additive (CI = 1) and antagonistic (CI > 1) effects of drug combination, respectively.

### 4.5. Real Time PCR

Total RNA was extracted by phenol-chloroform-ethanol method. RNA quantification was measured by absorption at 260 nm and the purity was determined by A260/A280 ratio. Then, 1 μg of RNA was converted into cDNA. And PCR was performed with SYBR Green PCR Master mix (TaKaRa Dalian Biotechnology Co., Ltd. Dalian, China) and primers (Table 2) by Fast 2500 Cyclers (Bio-Rad, Hercules, CA, USA).

### 4.6. Flow Cytometry

Cells were seeded with 3 × 10^5^ cells/well in six-well plates, treated with **LSS-11** (0.5, 2 μM) or the 0.1% DMSO vehicle for 24 h, cells were stained by Annexin V/PI for 15 min and detected by flowcytometry.

### 4.7. Immunofluorescence

A549/T cells were seeded with 3 × 10^5^ cells/well in 60 mm plates, and treated with **LSS-11** (0.5, 2 μM) or the 0.1% DMSO for 24 h, then fixed with 4% paraformaldehyde. Cells were incubated with primary antibodies for 2 h at room temperature, and exposure to FITC-linked secondary antibody for 1h at room temperature, then images were taken by confocal microscopy.

### 4.8. Immunoblotting Assay

Whole cell lysate (30 μg) were loaded and separated on 10% SDS-PAGE and transferred to PVDF membrane. Apoptotic associated proteins including DR5, PARP1, Bax, Bcl2, and GAPDH were incubated by corresponding antibodies and appropriate secondary anti-bodies and detected using chemiluminescent method.

### 4.9. Chromatin Immunoprecipitation (ChIP)

ChIP was performed according to the manufacturer’s instructions. Briefly, 6 × 10^6^ A549/T cells were fixed by 1.42% formaldehyde. STAT3-DNA was immunoprecipitated with anti-STAT3 antibody (Santa Cruz, #sc-482, 1 µg per 200 µg protein) overnight. Then anti-STAT3/STAT3/DNA complex was captured by protein A/G agarose beads (Thermo Fisher, Rockford, IL, USA), and reversely cross-linked, then DNA was purified by DNA column following manufacture’s instruction (Thermo Fisher, #26156). 50 µg cross-linked genomic DNA was used for qPCR to test with specific primers (Table 1) across MDR1 −198 bp to +43 bp around TSS promoter region and −504 bp~−398 bp of MRP1 promoter region.

### 4.10. Statistical Analysis

Values were shown as mean ± standard deviation, all the comparison between two groups was conducted by student’s *t* test with two-sided tail. The correlation between two groups was calculated by Pearson correlation coefficient. The significance was considered as *p* < 0.05. All the experiments performed at least in triplicates.

## 5. Conclusions

**LSS-11**, a novel triazolonaphthalimide-based topoisomerase inhibitor, overcomes paclitaxel-resistance in lung cancer cells via DR5/PARP1-mediated apoptosis and STAT3-mediated downregulation of MDR1 and MRP1. Our findings suggest an MDR1 downregulator rather than P-gp inhibitor can overcome paclitaxel resistance, and reveals triazolonaphthalimide as a novel agent for overcoming paclitaxel resistance.

## Figures and Tables

**Table 1:** IC_50_ values of paclitaxel and **LSS-11** in A549 and A549/T cells.

Drugs	A549 (μM)	A549/T (μM)	Resistance Index
Paclitaxel	1.9 ± 0.36	36.32 ± 8.29	19
LSS-11	>10	6.87 ± 0.77	~0.6

**Table 2:** Gene specific primer sequences used in qPCR assay.

Gene Symbol	Accession Number ^1^	Forward Primer Sequence	Reverse Primer Sequence
TOP2A	NM_001067.3	5′-GACGCTTCGTTATGGGAAGATA-3′	5′- GGGCCAGTTGTGATGGATAA -3′
MDR1	NM_001348946.1	5′-CAGCTATTCGAAGAGTGGGC-3′	5′-CCTGACTCACCACACCAATG-3′
MRP1	NM_004996.3	5′-ACCAAGACGTATCAGGTGGC-3′	5′-CTGTCAGGTTCCAGCTCCTC-3′
MRP2	NM_000392.4	5′-GCAGCGATTTCTGAAACACA-3′	5′-CAACAGCCACAATGTTGGTC-3′
MRP3	NM_003786.3	5′-CGCACACCGGCTTAACACTATCATGG-3′	5′-AAACCAGGAAAGGCCAGGAGGAAATC-3′
MRP4	NM_001301829.1	5′-GAGTTGCAAGGGTTCTGGGA-3′	5′-AAAGTCAGCACCGTGGCATA-3′
RPS18	NM_022551.2	5′-GATATGCTCATGTGGTGTTG-3′	5′-AATCTTCTTCAGTCGCTCCA-3′
MDR1-promoter	-	5′-GCAAGCTTCTAGAGAGGTGCAAC-3′	5′-AAAAGCTTGCGGCCTCTG-3′
MRP1-promoter	-	5′-TCTGTGTGACTCAGCTTTGG-3′	5′-GTGCAGAGAGGTTGAGTGATT-3′

^1^ Accession number from GeneBank.

## Supplementary Materials

The following are available online. Scheme 1: Synthesis scheme of **LSS-11**, Figure S1: Cell proliferation inhibition of A549 cells in the presence of **LSS-11** measured by MTT method, Figure S2: **LSS-11** has no effect on P-gp transport function, Figure S3: Heatmap depicting drug resistance genes in paclitaxel sensitive and insensitive A549 cells, Figure S4: **LSS-11** has no effect on transcription factors of MRP1 and MDR1.
