# Supplementary material for: The Novel Triazolonaphthalimide Derivative LSS-11 Synergizes the Anti-Proliferative Effect of Paclitaxel via STAT3-Dependent MDR1 and MRP1 Downregulation in Chemoresistant Lung Cancer Cells
Source: Molecules. 2017 Oct 26;22(11):1822. doi: 10.3390/molecules22111822 (PMC6150343; doi:10.3390/molecules22111822)
Supplement: Supplementary file 1 [file molecules-22-01822-s001.pdf]

Supplementary Materials:

The Novel Triazolonaphthalimide Derivative LS-111 Synergizes the Anti-proliferative Effects of Paclitaxel via STAT3-dependent MDR1/MRP1 Downregulation in Chemoresistant Lung Cancer Cells

Liyan Ji [1,2,3], Xi Liu [1], Shuwei Zhang [1], Shunan Tang [3], Simin Yang [3], Shasha Li [3], Xiaoxiao Qi [1], Siwang Yu [3], Linlin Lu [1], Xiangbao Meng [3,*] and Zhongqiu Liu [1,*]

1 International Institute for Translational Chinese Medicine, Guangzhou University of Chinese Medicine, Guangzhou 510006, China; liyanji@gzucm.edu.cn (L.J.); liuxi20150710@163.com (X.L); zswatobe@163.com (S.Z.); qixiaoxiao@gzucm.edu.cn (X.Q.); lllu@gzucm.edu.cn (L.L.)

2 The Postdoctoral Research Station, Guangzhou University of Chinese Medicine, Guangzhou 510006, China

3 Department of Chemical Biology, School of Pharmaceutical Sciences, Peking University, Beijing 100191, China; tangshunan1990@126.com (S.T.); yangsimin16@163.com (Sim.Y.); shashali@umich.edu (S.L.); swang_yu@bjmu.edu.cn (Siw.Y.)

* Correspondence: xbmeng@bjmu.edu.cn (X.M.); liuzq@gzucm.edu.cn (Z.L.); Tel.: +86-10-8280-1714 (X.M.); +86-020-3935-8061 (Z.L.)

Rhodamine 123 efflux assay

Cells were seeded with 3 × 10^5 cells/well in six-well plates, treated with LSS11 (0.5, 2 μM) or the 0.1% DMSO vehicle for 24h, then re-suspended in PBS containing Rho123 (final concentration, 1 μM). After 60 min of incubation at 37 °C, the cells were washed twice with cold PBS. And fluorescence intensity of rhodamine 123 was measured by a BD FACS Aria III flowcytometry (BD Biosciences, San Jose, CA, USA) at 488 nm argon laser and FL1 channel. All the data were analyzed by the Flow Jo 7.6.1 software (Tree Star, Inc., Ashland, OR, USA).

Procedure for the Synthesis of ***LSS-11***

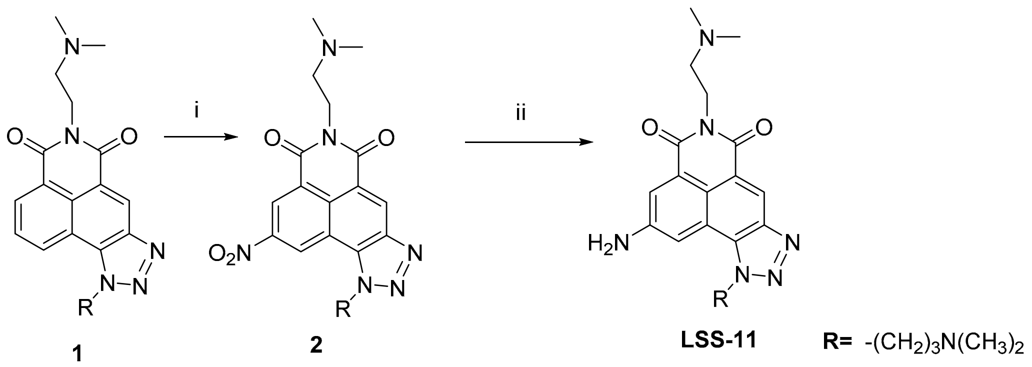

Scheme S1. Synthesis scheme of **LSS-11**. *Reagents and conditions*: (i) HNO_3 (65%), conc. H_2SO_4, 80°C; (ii) Pd/C, Et_3N, HCOOH, EtOH, 80°C.

Step 1

Compound **1** (100 mg) was dissolved in 1 mL of conc. H_2SO_4, then 65% HNO_3 (4 equiv.) was added. After stirring for 5 h at 80°C, the mixture was slowly diluted with 10 mL of H_2O, and neutralized with Na_2CO_3 until pH=8. The solution was extracted with CH_2Cl_2 three times, then the organic layers were combined, concentrated and purified by column chromatography to get a light yellow solid in 68% yield.

Step 2

Compound **2** (150 mg) was dissolved in 10 mL of EtOH, then Et_3N (0.25 mL), HCOOH (65 μL) and 10% Pd/C (20 mg) were added. The mixture was stirred for 2 h at 80°C, and a crystalline solid formed. The reaction solution was filtered after cooled it down and the residue was washed with ether. The residue was redissolved in CH_2Cl_2/MeOH (1:1), and filtered to remove Pd/C. The filtrate was concentrated and the solid recrystallized from ethanol to get a yellow solid in 95% yield. M.p. 234-235°C. ^{1}H-NMR (400 MHz, $CDCl_3/CD_3OD$) δ 8.63 (s, 1H), 8.01 (d, *J* = 2.1 Hz, 1H), 7.74 (d, *J* = 2.1 Hz, 1H), 5.02 (t, *J* = 6.8 Hz, 2H), 4.28 (t, *J* = 6.8 Hz, 2H), 2.69 (t, *J* = 6.7 Hz, 2H), 2.51 (d, *J* = 6.7 Hz, 2H), 2.34 (m, 14H). ^{13}C-NMR (101 MHz, $CDCl_3/CD_3OD$) δ 163.93, 163.69, 148.27, 143.57, 130.30, 123.84, 120.07, 120.03, 119.34, 119.21, 119.05, 108.88, 56.15, 55.78, 48.64, 44.80(2C), 44.68(2C), 37.54, 26.96. HR-ESI-MS: Calcd for $C_{21}H_{27}N_7O_2$ $[M+1]^+$: 410.22262; Found: 410.23004.

Supplementary Figures

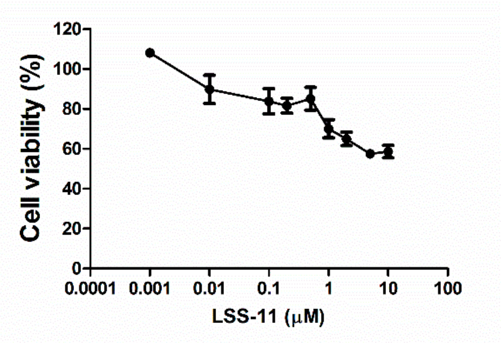

Figure S1. Cell proliferation inhibition of A549 cells in the presence or absence of **LSS-11** measured by MTT method.

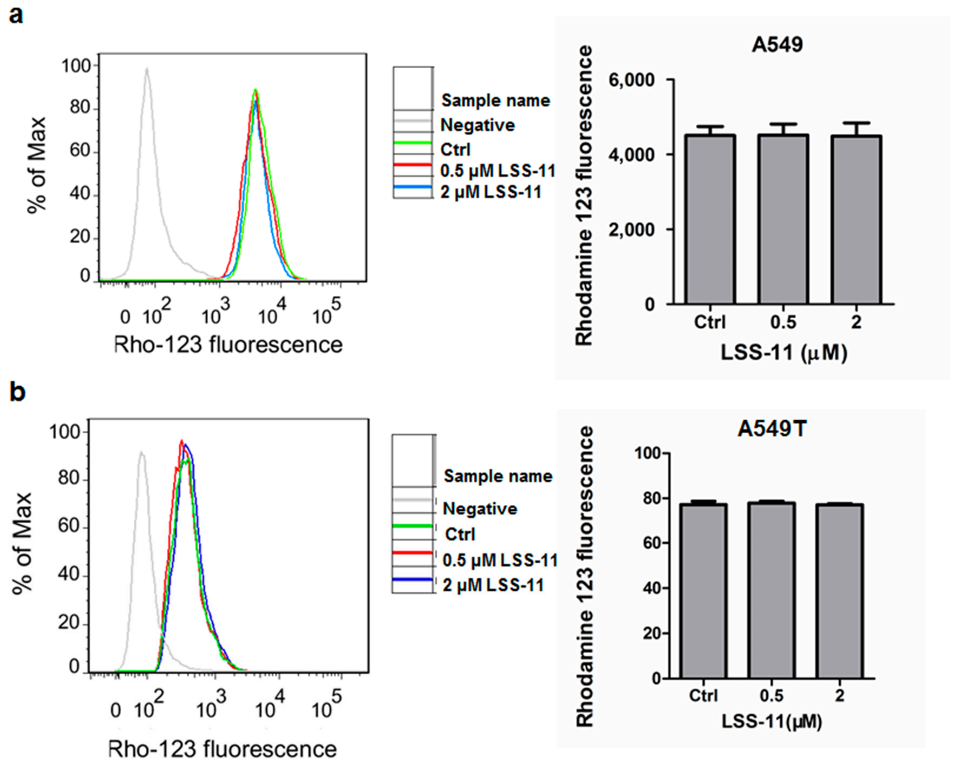

Figure S2. LSS-11 has no effect on p-gp transport function. Flow cytometry analysis of rhodamine 123 efflux in the presence of **LSS-11** in A549 (a) and A549/T (b) cells. Fluorescence intensity of rhodamine 123 shown as bar graph in A549 cells treated with **LSS-11** or vehicle (0.1 % DMSO). All data were obtained from three independent experiments.

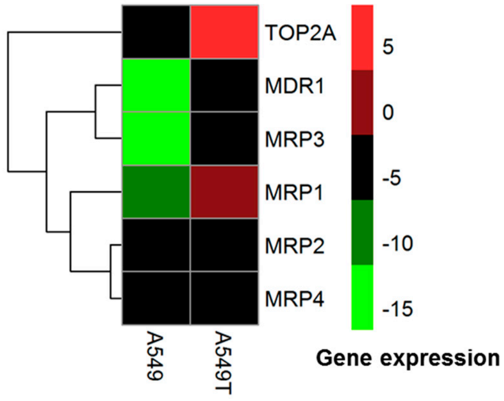

Figure S3. Heatmap depicting drug resistance genes in paclitaxel sensitive and insensitive A549 cells. mRNA levels of drug resistance genes in A549 and A549/T cells were detected by qPCR. Coding color indicates fold change of multiple drug resistance gene and monoresistance gene.

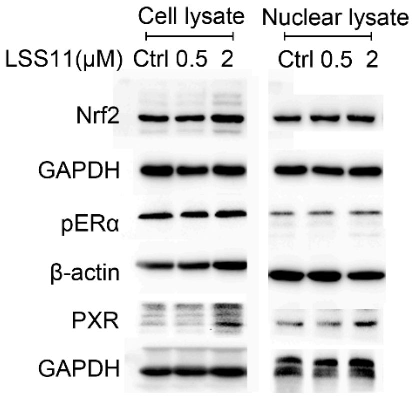

Figure S4. LSS-11 has no effect on transcription factors of MRP1 and MDR1. Western blot analysis of Nrf2, pERα, and PXR. GAPDH or β-actin were used as loading control.
